# A global cross-sectional survey on neonatal analgosedation: unveiling global trends and challenges through latent class analysis

**DOI:** 10.1007/s00431-025-06074-z

**Published:** 2025-03-12

**Authors:** Cristina Arribas, Giacomo Cavallaro, Nunzia Decembrino, Juan Luis González, Carolina Lagares, Genny Raffaeli, Anne Smits, Sinno P.H. Simons, Eduardo Villamor, Karel Allegaert, Felipe Garrido, on behalf of the ESPR Special Interest Group for Neonatal Pain

**Affiliations:** 1 Neonatal Intensive Care Unit, Clínica Universidad de Navarra, Madrid, Spain; 2 Neonatal Intensive Care Unit, Fondazione IRCCS Ca’ Granda Ospedale Maggiore Policlinico, Milan, Italy; 3 Neonatal Intensive Care Unit, Azienda Ospedaliera Universitaria Policlinico G. Rodolico San Marco, Catania, Italy; 4 Department of Statistics and Operations Research, Faculty of Medicine, University of Cadiz, Cádiz, Spain; 5 Department of Clinical Sciences and Community Health, Università Degli Studi Di Milano, Milan, Italy; 6 Department of Development and Regeneration, KU Leuven, Leuven, Belgium; 7 Neonatal Intensive Care Unit, University Hospitals Leuven, Leuven, Belgium; 8 Department of Pediatric and Neonatal Intensive Care, Division of Neonatology, Erasmus University Medical Center, Sophia Children’s Hospital, Rotterdam, The Netherlands; 9 Division of Neonatology, MosaKids Children’s Hospital, Maastricht University Medical Center (MUMC+), Research Institute for Oncology and Reproduction (GROW), Maastricht University, Maastricht, The Netherlands; 10 Department of Hospital Pharmacy, Erasmus MC, Rotterdam, The Netherlands; 11 Department of Pharmaceutical and Pharmacological Sciences, KU Leuven, Leuven, Belgium

**Keywords:** Newborn, Analgosedation, Tracheal intubation, Latent class analysis, Sociodemographic index

## Abstract

**Purpose:**

This study aims to analyze global prescribing patterns for analgosedation in neonates during four critical care scenarios. The research explores existing patterns, their association with geographic and sociodemographic index (SDI), and adherence to evidence-based practices.

**Methods:**

Data from a 2024 global survey of 924 responses to 28 questions were analyzed, focusing on four items for their high variability: premedication in intubation (Q17), sedation in preterm (Q19) and full-term newborns (Q23), and perinatal asphyxia (Q26). Latent class analysis (LCA) classified neonatal intensive care unit (NICU) prescriptions into patterns, assigning participants to the most likely class. Demographic variables, including geographic region and SDI, were compared using chi-square tests to assess associations.

**Results:**

Three distinct prescribing patterns emerged for each scenario. In premedication during intubation, Europe and North America predominantly used Class 1, adhering to guidelines with fentanyl, atropine, and muscle relaxants. In contrast, Class 2, standard in Asia and Latin America-Caribbean, primarily utilized fentanyl and midazolam, with rare use of atropine and muscle relaxants. For analgosedation in newborns, higher-SDI NICUs favored fentanyl, while lower-SDI NICUs preferred midazolam or morphine combinations. In perinatal asphyxia cases, fentanyl was the leading choice in Class 3, especially in Europe. Dexmedetomidine use was limited, primarily appearing in Class 1 NICUs.

**Conclusion:**

The study highlights substantial regional variability in neonatal analgosedation, influenced by SDI and geography. Despite established guidelines, gaps in evidence-based implementation persist. These findings underscore the need for global standardization of neonatal care protocols and further research on the long-term safety of midazolam and dexmedetomidine.

**Table:** 

**What is Known:**	
• *Previous research has demonstrated significant disparities in prescribing patterns for neonatal analgosedation across geographic areas influenced by demographic and socioeconomic factors.* • *Midazolam remains a commonly utilized agent in neonatal analgosedation despite evidence suggesting potential neurodevelopmental risks, particularly in premature infants.* • *Current guidelines regarding neonatal analgesia and sedation, including premedication for endotracheal intubation, are not consistently implemented, particularly in regions characterized by lower sociodemographic indices.*	
**What is New:**	
• *This study employs Latent Class Analysis (LCA) to categorize global neonatal prescribing practices into three distinct patterns, elucidating regional differences and compliance with evidence-based guidelines.* • *Care providers working in countries with higher Sociodemographic Index (SDI) are more likely to adhere to evidence-based practices, such as intubation premedication, than regions with medium or medium–high SDI.* • *The use of midazolam in full-term and preterm newborns exposes a gap between evidence-based guidelines and clinical practices. This situation calls for more research on the long-term safety of midazolam and the development of standardized sedation protocols that emphasize safer alternatives to reduce associated risks in neonatal care.* • *Dexmedetomidine is underutilized globally despite its increasing applications, highlighting the need for more pharmacokinetic and pharmacodynamic research before its inclusion in clinical guidelines.*

**Supplementary Information:**

The online version contains supplementary material available at 10.1007/s00431-025-06074-z.

## Introduction

Infants admitted to the neonatal intensive care unit (NICU) undergo a variety of procedures that can lead to pain and stress. Recognizing and managing neonatal pain and agitation is a quality indicator of good clinical practice. Despite increased awareness of the significant impact of neonatal pain and distress on both short- and long-term outcomes, there remains considerable variability in analgesia and sedation management practices among NICUs globally, which is evident across different countries and units [1–3].

Understanding the various approaches to neonatal analgesia and sedation is essential for comprehending the rationale for their use and the potential for standardization based on scientific evidence. Additionally, it is necessary to consider how social and economic factors may influence clinical practices in neonatology. Our working group recently conducted a global cross-sectional survey documenting that countries’ socio-economic characteristics impact neonatal analgosedation management and that there are differences in neonatal pain management and sedation based on the sociodemographic index (SDI) [4].

In the present paper, we have applied latent class analysis (LCA) to the four most variable items of our previous global survey data in order to identify patterns in neonatal analgosedation management in the NICUs. LCA is a measurement model similar to confirmatory factor analysis (CFA), which assumes that the structure of a set of observable indicators results from latent variables, referred to as latent classes [5]. This technique enables the identification of subpopulations within a larger group that may not be apparent through conventional analytical approaches. In contrast to traditional “variable-centered” analyses, which examine the relationships among variables, LCA focuses on clustering individuals into distinctive latent classes, each characterized by analogous behaviors [6; 7].

The present analysis will specifically address four settings: premedication for intubation in both term and preterm newborns, the utilization of sedative drugs in preterm newborns, the administration of sedation during mechanical ventilation in term newborns, and the application of sedative drugs in newborns with perinatal asphyxia. This analysis comprehensively explains current practices and the variability in administering these drugs within different neonatal clinical environments.

### Methods

The data used in this analysis were extracted from our previous global, prospective, cross-sectional survey published in 2024 [4]. The original survey comprised 28 questions and was developed using a modified Delphi method. It can be consulted in our original paper [4; 8; 9].

Access to the original questionnaire was provided in English through the Survey Monkey platform (https://www.surveymonkey.com). The study was approved by the Ethics Committee of the University of Navarra (Spain; Code 2022.186). This approval covered both the information sheet for the participants and the questionnaire. Respondents were informed through the cover letter that participation in the survey was voluntary. The survey was distributed through neonatal or pediatric societies, working groups, authorized email lists, general and specific neonatal social networks (https://99nicu.org/), professionals (http://www.linkedin.com), and their networks. Staff contacted by the researchers were requested to avoid duplicate responses from the same NICU/hospital. Respondents were invited to voluntarily provide their center name to identify duplicate responses from the same NICU.

From the survey, we used four items from the original questionnaire, which addressed the following topics: use of premedication in intubation procedures (question 17), administration of sedative drugs in preterm newborns (question 19), use of sedation during mechanical ventilation in full-term newborns (question 23) and the sedation in newborns with perinatal asphyxia (question 26).

We have carried out a LCA using key survey questions as indicators. This model allowed us to classify NICU prescriptions into patterns characterized by indicators [6]. First, we explored the solution with one pattern of initial fit parameters. Then, we increased the number of classes until we obtained the best fit for the model. To evaluate the fit, we used several criteria. First, the Akaike Information Criteria (AIC), the Bayesian Information Criterion (BIC), and the Adjusted BIC, where small values of each indicate a better model fit [10–12]. Second, we employed the entropy value, which is a metric that ranges from 0 to 1 and quantifies the uncertainty associated with the classification of different classes. An entropy value exceeding 0.80 signifies a robust separation, effectively identifying distinct groups [13]. Third, we also used the Lo-Mendell-Rubin Adjusted Likelihood Ratio Test (LMRT) and the Bootstrapped Likelihood-Ratio Test (BLRT), suggesting that, for a p < 0.05, the model with more classes fits the data worse than the model with fewer classes [14]. In addition to the statistical fit, we also used the significance and interpretation of the patterns, as well as the probability of the presence of each indicator in the pattern [15]. To perform the LCA, we used the NICUs’ responses on the types of drugs used in the four scenarios examined and recorded as binary indicators. Once the profiles were obtained, we calculated everyone’s class membership probabilities. This allowed the identification of a categorical variable within categories by assigning the class with the highest membership probability to each participant.

We analyzed selected demographic variables from NICUs to compare the prescribing patterns derived from LCA. The variables are presented in Tables 1 to 4. The SDI was stratified into four categories in alignment with the original study. Furthermore, we employed the chi-square test to investigate the relationship between the latent classes or prescription patterns and the demographic variables.

**Table 1:** Premedication for endotracheal intubation

	TOTAL	Class 1	Class 2	Class 3	
	924 (100.0%)	273 (29.5%)	515 (55.7%)	136 (14.7%)	p
Continent (n = 924)					0,000
Africa	16 (1,7)	2 (0,7)	5 (0,9)	9 (6,6)	
Asia	150 (16,2)	21 (7,6)	113 (21,9)	16 (11,7)	
Europe	393 (42,5)	144 (52,7)	169 (32,8)	80 (58,8)	
Middle East	63 (6,8)	10 (3,6)	42 (8,1)	11 (8)	
North America	127 (13,7)	58 (21,2)	58 (11,2)	11 (8)	
Oceania	22 (2,3)	15 (5,4)	0 (0)	7 (5,1)	
Latin America and the Caribbean	153 (16,5)	23 (8,4)	128 (24,8)	2 (1,4)	
NICU level (n = 924)					0.023
Level 1 Neonatal Unit	14 (1,5)	2 (0,7)	8 (1,5)	4 (2,9)	
Level 2 Neonatal Unit	98 (10,6)	23 (8,4)	66 (12,8)	9 (6,6)	
Level 3 NICU	337 (36,4)	96 (35,1)	189 (36,6)	52 (38,2)	
Level 3 NICU with surgery	447 (48,3)	146 (53,4)	239 (46,4)	62 (45,5)	
Mixed pediatric and NICU	28 (3)	6 (2,1)	13 (2,5)	9 (6,6)	
NICU looking after less than 28 weeks (n = 923)					0.729
No	85 (9,2)	27 (9,9)	44 (8,5)	14 (10,2)	
Yes	838 (90,7)	245 (90)	471 (91,4)	122 (89,7)	
NICU looking after less than 1500 g (n = 914)					0.049
< 50	331 (36,2)	90 (33,5)	203 (39,5)	38 (28,5)	
50–100	325 (35,5)	90 (33,5)	180 (35)	55 (41,3)	
> 100	258 (28,2)	88 (32,8)	130 (25,3)	40 (30)	
NICU number of cots (n = 923)					0.085
< 10	266 (28,8)	75 (27,4)	154 (29,9)	37 (27,2)	
10–30	500 (54,1)	142 (52)	273 (53,1)	85 (62,5)	
> 30	157 (17)	56 (20,5)	87 (16,9)	14 (10,2)	
NICU having sedation/analgesia guidelines (n = 921)					0.000
No	372 (40,3)	82 (30)	238 (46,3)	52 (38,5)	
Yes	549 (59,6)	191 (69,9)	275 (53,6)	83 (61,4)	
SDI recodified 4 categories (n = 924)					0.000
Low & Low-Middle SDI	78 (8,4)	12 (4,3)	51 (9,9)	15 (11)	
Middle SDI	199 (21,5)	23 (8,4)	163 (31,6)	13 (9,5)	
High-Middle SDI	319 (34,5)	85 (31,1)	205 (39,8)	29 (21,3)	
High SDI	328 (35,4)	153 (56)	96 (18,6)	79 (58)	

Summary of demographic characteristics of participant NICUs in contrast with the prescription patterns obtained. The categorical variables are expressed as n (%). The statistical relationship between these categorical variables was calculated using the chi-square test, with values ​​of *p* < 0.05 considered statistically significant. *NC: not calculable. *NICU*: neonatal intensive care unit; *SDI*: sociodemographic index

We used Mplus software, version 8.10 (Muthén & Muthén, 3463 Stoner Avenue, Los Angeles, CA 90066, US), to perform LCA and SPSS, version 29 (IBM Corp., Armonk, NY 10504, US) to perform the descriptive analyses and assess the associations [16; 17]. In all analyses, we considered a p < 0.05 as statistically significant.

## Results

The study collected 924 responses from 98 countries, with a notable concentration from European NICUs at 42.5%, followed by Latin America and the Caribbean at 16.5%. Africa contributed the fewest responses, accounting for only 1.7%. Most NICUs were classified as level 3, with and without surgery, representing 84.7% of the total. Additionally, 90.7% of these units cared for newborns born at less than 28 weeks of gestational age. However, only 59.6% of the NICUs reported having pain management guidelines, and 69.9% were categorized as High-Middle and High SDI.

### Premedication for endotracheal intubation

**Fig. 1:**
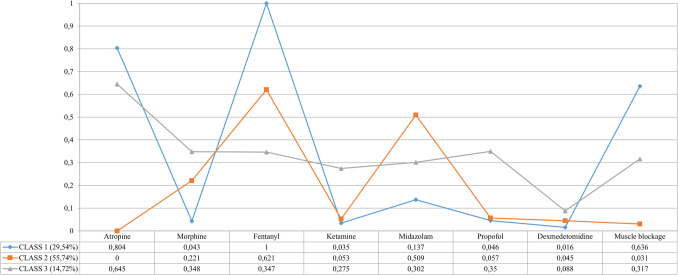
Graphical representation and data related to latent classes obtained with the use of analgosedation drugs for premedication in intubation

Analysis of data from LCAs revealed three distinct patterns of premedication used for tracheal intubation. Figure 1 displays the graphical representation of the latent class model.

Class 2 is the most common prescribing pattern, with 515 NICUs making up 55.7% of the data. The main agents for intubation premedication are fentanyl (62%) and midazolam (51%), followed by morphine (22%). Notably, atropine is absent (0%), and muscle relaxants (3%), ketamine (5%), and dexmedetomidine (4.5%) are rarely used. Class 2 also shows higher midazolam use than the other classes and has a significant proportion of NICUs in Asia and the Latin America-Caribbean region (46.7%). The predominant SDI for this class is medium (31.6%) and medium–high (39.8%).

Class 1 includes 273 NICUs, representing 29.5% of the dataset. This class exclusively uses fentanyl (100%) and frequently co-administers atropine (80.4%) and muscle relaxants (63.6%). Propofol and morphine are used minimally (less than 5%), while dexmedetomidine is used in less than 2% of cases. Most NICUs in Class 1 are located in Europe (52.7%) and North America (21.2%), with 87.1% falling within the medium–high to high SDI category.

Class 3 comprises 136 NICUs (14.7%) and is the least frequently observed pattern. It is characterized by higher uses of morphine (34.8%), ketamine (27.5%), propofol (35%), and atropine (64.5%). Muscle relaxants are utilized in about one in three NICUs (31.7%), while dexmedetomidine usage is higher in this class (8.8%) compared to Classes 1 (1.6%) and 2 (4.5%).

This class mainly includes NICUs from Europe (58.8%) and Asia (11.7%), with a significant representation of countries with high (58%) and medium–high (21.3%) SDI ratings, as well as a notable proportion of low SDI countries (11%) compared to Class 1 (4.3%) and Class 2 (9.9%).

Table 1 summarizes these results and analyzes the relationship between patterns and demographic variables in NICUs, showing a specific statistical significance.

### Use of analgesic and sedative drugs in preterm newborns

**Fig. 2:**
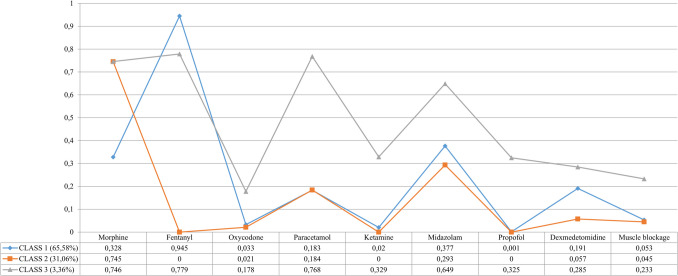
Graphical representation and data related to latent classes obtained with the use of analgosedation drugs in preterm newborns

The LCA model was applied to identify three distinct patterns concerning the administration of analgesia and sedation in preterm infants. The graphical representation of the latent class model is illustrated in Fig. 2.

Class 1, comprising 606 NICUs (65.6%), predominantly uses fentanyl (94.5%), midazolam (37.7%), and morphine (32.8%). In contrast, Class 2 includes 287 NICUs (31.1%) and primarily utilizes morphine (74.5%) and midazolam (29.3%). Neither class prescribes oxycodone, ketamine, or propofol, and a third pattern accounts for only 3.4% of NICUs.

Class 1 NICUs are mainly in Europe (41.2%) and Latin America-Caribbean (20.6%), distributed across various SDI categories: high-middle (41.4%), middle (24.5%), and high (25.5%). Class 2 units are primarily found in Europe (44.2%) and Asia (18.4%), with 56.4% classified as high SDI.

Table 2 summarizes these results and examines the relationship between prescribing patterns and demographic variables in NICUs, demonstrating specific patterns of association.

**Table 2:** Use of sedation and analgesia drugs in preterm newborns

	Total	Class 1	Class 2	Class 3	
	924 (100,0%)	606 (65.6%)	287 (31.1%)	31 (3.4%)	p
Continent (n = 924)					NC*
Africa	16 (1,7)	8 (1,3)	8 (2,7)	0 (0)	
Asia	150 (16,2)	91 (15)	53 (18,4)	6 (19,3)	
Europe	393 (42,5)	250 (41,2)	127 (44,2)	16 (51,6)	
Middle East	63 (6,8)	38 (6,2)	22 (7,6)	3 (9,6)	
North America	127 (13,7)	83 (13,6)	41 (14,2)	3 (9,6)	
Oceania	22 (2,3)	11 (1,8)	9 (3,1)	2 (6,4)	
Latin America and the Caribbean	153 (16,5)	125 (20,6)	27 (9,4)	1 (3,2)	
NICU level (n = 924)					0.235*
Level 1 Neonatal Unit	14 (1,5)	6 (0,9)	8 (2,7)	0 (0)	
Level 2 Neonatal Unit	98 (10,6)	59 (9,7)	38 (13,2)	1 (3,2)	
Level 3 NICU	337 (36,4)	226 (37,2)	101 (35,1)	10 (32,2)	
Level 3 NICU with surgery	447 (48,3)	296 (48,8)	132 (45,9)	19 (61,2)	
Mixed pediatric and NICU	28 (3)	19 (3,1)	8 (2,7)	1 (3,2)	
NICU looking after less than 28 weeks (n = 923)					0.446
No	85 (9,2)	55 (9)	29 (10,1)	1 (3,2)	
Yes	838 (90,7)	550 (90,9)	258 (89,8)	30 (96,7)	
NICU looking after less than 1500 g (n = 914)					0.042
< 50	331 (36,2)	233 (38,7)	90 (31,9)	8 (26,6)	
50–100	325 (35,5)	218 (36,2)	95 (33,6)	12 (40)	
> 100	258 (28,2)	151 (25)	97 (34,3)	10 (33,3)	
NICU number of cots (n = 923)					0.200
< 10	266 (28,8)	182 (30)	80 (27,8)	4 (12,9)	
10–30	500 (54,1)	326 (53,8)	152 (52,9)	22 (70,9)	
> 30	157 (17)	97 (16)	55 (19,1)	5 (16,1)	
NICU having sedation/analgesia guidelines (n = 921)					0.081
No	372 (40,3)	260 (42,9)	102 (35,7)	10 (32,2)	
Yes	549 (59,6)	345 (57)	183 (64,2)	21 (67,7)	
SDI recodified 4 categories (n = 924)					0.000
Low & Low-Middle SDI	78 (8,4)	51 (8,4)	25 (8,7)	2 (6,4)	
Middle SDI	199 (21,5)	149 (24,5)	44 (15,3)	6 (19,3)	
High-Middle SDI	319 (34,5)	251 (41,4)	56 (19,5)	12 (38,7)	
High SDI	328 (35,4)	155 (25,5)	162 (56,4)	11 (35,4)	

Summary of demographic characteristics of participant NICUs in contrast with the prescription patterns obtained. The categorical variables are expressed as n (%). The statistical relationship between these categorical variables was calculated using the chi-square test, with values ​​of *p *< 0.05 considered statistically significant. *NC: not calculable. *NICU*: neonatal intensive care unit; *SDI*: sociodemographic index

### Use analgesic and sedative drugs during mechanical ventilation in full-term newborns

**Fig. 3:**
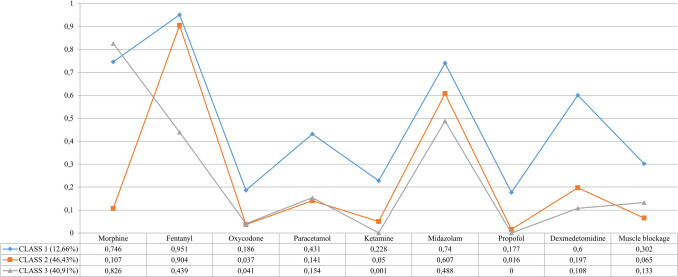
Graphical representation and data related to latent classes obtained using sedation and analgesia drugs during mechanical ventilation in term newborns

LCA has identified a superior model consisting of three distinct patterns of analgesia and sedation use during mechanical ventilation in term newborns. Figure 3 provides a graphical representation of this latent class model. The predominant patterns are Class 2, which comprises 429 NICUs (46.4%), and Class 3, including 378 NICUs (40.9%).

In Class 2, the primary medications used are fentanyl (90.4%) and midazolam (60.7%). Other pharmacological agents are utilized somewhat, and this class shows the lowest incidence of muscle relaxant use (6.5%). Notably, morphine administration in this class is only 10.7%, significantly lower than the rates observed in Class 1 (74.6%) and 3 (82.6%). This pattern is prevalent in Europe (43.1%) and Asia (18.6%), making it the most prevalent in countries with a medium–high SDI (44.9%).

Class 3 is characterized by the highest utilization of morphine (82.6%) and the lowest use of fentanyl (43.9%). In this class, propofol is not used, and ketamine administration is less than 1%. This pattern is predominantly found in countries with a high SDI (52.3%).

Class 1, the least frequent, accounts for 12.7% of NICUs and reports the highest usage of dexmedetomidine (60%) compared to Class 2 (19.7%) and Class 3 (10.8%). Additionally, Class 1 has the highest incidence of muscle relaxant use (30.2%).

Table 3 summarizes the findings and analyzes relationships between patterns and demographic variables in NICUs, revealing statistically significant results.

**Table 3:** Use of sedation and analgesia drugs during mechanical ventilation in term newborns

	Total	Class 1	Class 2	Class 3	
	924 (100,0%)	117 (12.7%)	429 (46.4%)	378 (40.9%)	p
Continent (n = 924)					0.000
Africa	16 (1,7)	1 (0,8)	4 (0,9)	11 (2,9)	
Asia	150 (16,2)	8 (6,8)	80 (18,6)	62 (16,4)	
Europe	393 (42,5)	63 (53,8)	185 (43,1)	145 (38,3)	
Middle East	63 (6,8)	5 (4,2)	33 (7,6)	25 (6,6)	
North America	127 (13,7)	21 (17,9)	51 (11,8)	55 (14,5)	
Oceania	22 (2,3)	8 (6,8)	0 (0)	14 (3,7)	
Latin America and the Caribbean	153 (16,5)	11 (9,4)	76 (17,7)	66 (17,4)	
NICU level (n = 924)					0.000
Level 1 Neonatal Unit	14 (1,5)	1 (0,8)	8 (1,8)	5 (1,3)	
Level 2 Neonatal Unit	98 (10,6)	4 (3,4)	57 (13,2)	37 (9,7)	
Level 3 NICU	337 (36,4)	25 (21,3)	170 (39,6)	142 (37,5)	
Level 3 NICU with surgery	447 (48,3)	84 (71,7)	175 (40,7)	188 (49,7)	
Mixed pediatric and NICU	28 (3)	3 (2,5)	19 (4,4)	6 (1,5)	
NICU looking after less than 28 weeks (n = 923)					0.043
No	85 (9,2)	5 (4,3)	49 (11,4)	31 (8,2)	
Yes	838 (90,7)	111 (95,6)	380 (88,5)	347 (91,7)	
NICU looking after less than 1500 g (n = 914)					0.002
< 50	331 (36,2)	34 (30)	179 (41,9)	118 (31,5)	
50–100	325 (35,5)	43 (38)	152 (35,5)	130 (34,7)	
> 100	258 (28,2)	36 (31,8)	96 (22,4)	126 (33,6)	
NICU number of cots (n = 923)					0.000
< 10	266 (28,8)	14 (11,9)	153 (35,7)	99 (26,1)	
10–30	500 (54,1)	84 (71,7)	206 (48,1)	210 (55,5)	
> 30	157 (17)	19 (16,2)	69 (16,1)	69 (18,2)	
NICU having sedation/analgesia guidelines (n = 921)					0.662
No	372 (40,3)	46 (39,3)	180 (41,9)	146 (38,9)	
Yes	549 (59,6)	71 (60,6)	249 (58)	229 (61)	
SDI recodified 4 categories (n = 924)					0.000
Low & Low-Middle SDI	78 (8,4)	2 (1,7)	47 (10,9)	29 (7,6)	
Middle SDI	199 (21,5)	19 (16,2)	113 (26,3)	67 (17,7)	
High-Middle SDI	319 (34,5)	42 (35,8)	193 (44,9)	84 (22,2)	
High SDI	328 (35,4)	54 (46,1)	76 (17,7)	198 (52,3)	

Summary of demographic characteristics of participant NICUs in contrast with the prescription patterns obtained. The categorical variables are expressed as n (%). The statistical relationship between these categorical variables was calculated using the chi-square test, with values ​​of *p *< 0.05 considered statistically significant. *NICU*: neonatal intensive care unit; *SDI*: sociodemographic index

### Use of analgesic and sedative drugs in newborns with perinatal asphyxia

**Fig. 4:**
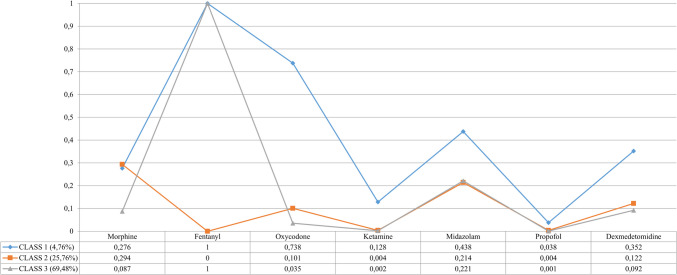
Graphical representation and data related to latent classes obtained with the use of analgesia or sedation in asphyxiated neonates undergoing therapeutic hypothermia

Three distinct patterns were identified through LCA regarding the application of analgesia and sedation in neonates experiencing perinatal asphyxia. The graphical result of the latent class model obtained is represented in Fig. 4.

Class 3, comprising 642 NICUs (69.5%), is the largest category identified. Fentanyl was used in 100% of cases, while midazolam was used in 22.1%. Other medications accounted for less than 10%. This class is mainly represented by NICUs in Europe (46.1%), followed by Latin America-Caribbean (17.1%) and Asia (14.3%).

Class 2 includes 238 NICUs (25.8%), with morphine used in 29.4% of cases and midazolam in 21.4%. Notably, fentanyl was not utilized in this category. Class 2 follows an almost uniform distribution in the 4 SDI categories, with significant representation from NICUs in Europe (30.6%), Asia (23.5%), and North America (21.4%).

Class 1, the least frequent, includes 44 NICUs (4.8%). Fentanyl was reported in 100% of cases, followed by oxycodone (73.8%), midazolam (43.8%), dexmedetomidine (35.2%), and morphine (27.6%). This class is primarily represented by NICUs in Europe (54.5%) and North America (15.9%). Table 4 summarizes these results and examines the relationship between NICUs’ medication use and demographic variables.

**Table 4:** Use analgesia or sedation in asphyxiated neonates undergoing therapeutic hypothermia

	Total	Class 1	Class 2	Class 3	
	924 (100,0%)	44 (4.8%)	238 (25.8%)	642 (69.5%)	p
Continent (n = 924)					0.000
Africa	16 (1,7)	2 (4,5)	10 (4,2)	4 (0,6)	
Asia	150 (16,2)	2 (4,5)	56 (23,5)	92 (14,3)	
Europe	393 (42,5)	24 (54,5)	73 (30,6)	296 (46,1)	
Middle East	63 (6,8)	3 (6,8)	9 (3,7)	51 (7,9)	
North America	127 (13,7)	7 (15,9)	51 (21,4)	69 (10,7)	
Oceania	22 (2,3)	1 (2,2)	1 (0,4)	20 (3,1)	
Latin America and the Caribbean	153 (16,5)	5 (11,3)	38 (15,9)	110 (17,1)	
NICU level (n = 924)					NC*
Level 1 Neonatal Unit	14 (1,5)	0 (0)	6 (2,5)	8 (1,2)	
Level 2 Neonatal Unit	98 (10,6)	5 (11,3)	47 (19,7)	46 (7,1)	
Level 3 NICU	337 (36,4)	12 (27,2)	82 (34,4)	243 (37,8)	
Level 3 NICU with surgery	447 (48,3)	25 (56,8)	92 (38,6)	330 (51,4)	
Mixed pediatric and NICU	28 (3)	2 (4,5)	11 (4,6)	15 (2,3)	
NICU looking after less than 28 weeks (n = 923)					0.000
No	85 (9,2)	1 (2,2)	42 (17,6)	42 (6,5)	
Yes	838 (90,7)	43 (97,7)	196 (82,3)	599 (93,4)	
NICU looking after less than 1500 g (n = 914)					0.000
< 50	331 (36,2)	18 (41,8)	114 (48,3)	199 (31,3)	
50–100	325 (35,5)	15 (34,8)	61 (25,8)	249 (39,2)	
> 100	258 (28,2)	10 (23,2)	61 (25,8)	187 (29,4)	
NICU number of cots (n = 923)					0.016
< 10	266 (28,8)	11 (25)	87 (36,5)	168 (26,2)	
10–30	500 (54,1)	26 (59)	107 (44,9)	367 (57,2)	
> 30	157 (17)	7 (15,9)	44 (18,4)	106 (16,5)	
NICU having sedation/analgesia guidelines (n = 921)					0.126
No	372 (40,3)	14 (31,8)	107 (45,3)	251 (39,1)	
Yes	549 (59,6)	30 (68,1)	129 (54,6)	390 (60,8)	
SDI recodified 4 categories (n = 924)					0.001
Low & Low-Middle SDI	78 (8,4)	2 (4,5)	29 (12,1)	47 (7,3)	
Middle SDI	199 (21,5)	9 (20,4)	71 (29,8)	119 (18,5)	
High-Middle SDI	319 (34,5)	16 (36,3)	67 (28,1)	236 (36,7)	
High SDI	328 (35,4)	17 (38,6)	71 (29,8)	240 (37,3)	

Summary of demographic characteristics of participant NICUs in contrast with the prescription patterns obtained. The categorical variables are expressed as n (%). The statistical relationship between these categorical variables was calculated using the chi-square test, with values ​​of *p *< 0.05 considered statistically significant. *NC: not calculable. *NICU*: neonatal intensive care unit; *SDI*: sociodemographic index

## Discussion

This article outlines prescription patterns for four stressful neonatal procedures: endotracheal intubation, mechanical ventilation for premature and full-term newborns, and therapeutic hypothermia, based on our previous study on global analgosedation drug use [4].

In this secondary study, we enhance our contributions by providing an alternative interpretation of the results using LCA, a statistical method that identifies unobservable subgroups in a population based on observed data. This method is valuable in health studies for uncovering hidden patterns in complex data and has been used in neonatology [6; 7; 16; 18–20]. Spezia et al. used the method to investigate parental experiences in neonatal units [21]. Momany et al. employed it to identify risk factors affecting neonatal neurodevelopment [22]. Ullsten et al. applied LCA to categorize NICUs based on parental involvement in managing neonatal pain [23].

Two main patterns of premedication for endotracheal intubation are observed. The first involves fentanyl, atropine, and muscle relaxants. The second feature is either fentanyl or midazolam alone, often without medications to prevent bradycardia, particularly in countries with medium to medium–high SDI. In 2010, the American Academy of Pediatrics recommended premedication for neonatal intubations, except in emergency resuscitation, using medications from prescription pattern 1 [24]. Recent literature highlights a link between prescription patterns based on evidence and healthcare units in higher sociodemographic areas. A meta-analysis also shows that premedication for neonatal intubation increases safety by reducing vital signs fluctuations and pain, ultimately improving the procedure’s overall conditions [25]. Moreover, midazolam enhances intubation conditions when combined with short-action analgesics, such as fentanyl or remifentanil, but is not suitable alone or for preterm neonates under 34 weeks due to side effects [26; 27]. Morphine has a slower onset and longer half-life, resulting in less effective analgesia during the procedure and an increased risk of later adverse events, like apnea [26].

The use of analgosedation in premature newborns showed two patterns: one more frequent, which mainly prescribes fentanyl, and another that uses primarily morphine. These were the two expected scenarios, with the association of midazolam use in premature newborns being observed in both patterns. Carbajal et al., in the EUROPAIN study, also confirmed this fact and showed that midazolam was the most commonly used sedative drug in European NICUs [28]. Clinical and preclinical studies have documented an increase in the deleterious effect on neurodevelopment in premature infants treated with midazolam, so, surprisingly, this sedative continues to play such a relevant role in the prescription of sedoanalgesia in this group of newborns [27; 29; 30]. In a recent review, Ancora et al. emphasize that both fentanyl and remifentanil should be used before painful procedures instead of morphine, especially in situations of risk of hypotension or gestational age less than 27 weeks [30]. However, Ziesenitz et al. warn that the use of fentanyl in premature infants shows a high variability in its pharmacokinetics, whether used as a bolus or as continuous infusion. They also highlight the ability to cause iatrogenic withdrawal symptoms a few days after the start of its use [31]. Nevertheless, consistent weaning protocols can effectively alleviate this issue [32].

Regarding the use of analgesia and sedation in full-term newborns, we also obtained two patterns similar to those found in premature newborns. One pattern highlighted the administration of fentanyl, while the other focused on morphine. Both patterns were found to be associated with midazolam. In the first pattern, a reduction in the use of muscle relaxants was noted. On a global scale, the similarities in the frequency of these patterns suggest that there is no definitive preference for either morphine or fentanyl. As noted by McPherson et al., severe conditions in full-term neonates frequently require continuous infusion of analgesics and sedatives, thereby allowing for an interchangeable use of fentanyl and morphine, either alone or in combination with midazolam, even if midazolam should be avoided for its adverse effects on neurodevelopment [27; 29].

Concerning analgosedation during therapeutic hypothermia, the most frequent prescription pattern included the use of fentanyl and midazolam. The second most common pattern reported the use of morphine and midazolam. While therapeutic hypothermia in neonates can cause stress and pain, there is no consensus on the use of analgosedation, and research assessing these aspects is limited [34; 35]. While some studies on therapeutic hypothermia do not mention the use of opioids for sedation, Eicher et al. suggested using morphine or fentanyl in cases of cardiovascular instability [35–38]. The potential neuroprotective effects of morphine administration during hypothermia have been questioned by findings from the MARBLE study [39]. It is striking that dexmedetomidine is rarely present in the two most frequent patterns, contrary to what was observed by Stark et al., who have recently highlighted a notable relative increase in its use from 2010 to 2018 in NICUs [40]. While it is not FDA-approved for neonates and lacks sufficient evidence for routine use in mechanical ventilation in newborns, it seems to have a favorable safety profile in term neonates, particularly during cardiopulmonary bypass [42; 43]. Pharmacokinetic studies suggest low adverse effects, supporting its increasing use in NICU, even for minimally invasive procedures such as the less invasive surfactant administration (LISA) technique [44; 45]. This alpha-2 adrenergic agonist seems to induce some neuroprotection in animal models, although its effects in newborns are less robust. McPherson et al. highlight the lack of induction of hypotension and the reduction in the time to start enteral feeding in hypothermia patients [45].

This study has several limitations that should be considered. First, its cross-sectional design captures prescribing practices at a single time, preventing the assessment of trends and changes over time. Second, the data were self-reported, making the study susceptible to recall and response biases that could affect its accuracy. Third, despite efforts to ensure global representation, the responses were uneven, with some geographic regions being underrepresented. This limits the generalizability of our findings, particularly in lower SDI regions.

Fourth, while the LCA approach provided a novel classification of prescribing patterns, it was based solely on reported practices rather than clinical outcomes. This prevents a direct evaluation of the effectiveness or safety of different sedation strategies. Additionally, variability in national guidelines, institutional protocols, and resource availability may have influenced the responses, introducing unforeseen confounding factors.

We also did not assess other factors that may shape prescribing behavior, such as training programs, staff experience, or the availability of specific medications. Lastly, the study did not analyze the impact of sedation practices on neonatal outcomes, emphasizing the need for future prospective studies that link prescribing patterns with clinical results.

Pain exposure has long-term adverse effects on pain processing, cognition, behavior, and neurodevelopment. Despite this, pain is often undertreated, and treatment practices vary widely. This publication offers a statistical analysis of neonatal analgosedation prescription patterns worldwide. Our findings highlight gaps in care provision, including harmful practices, and provide insights to guide improvements and research. The goal is to enhance understanding of prescribing trends and identify opportunities for further studies across different areas.

## Conclusions

In conclusion, LCA revealed distinct global neonatal analgesia and sedation patterns shaped by geography and SDI. Fentanyl is the most common medication, but its combinations vary significantly. Despite recommendations for comprehensive premedication in intubation, medium to medium–high SDI regions often omit adjuvant drugs. The ongoing use of midazolam in preterm infants raises concerns about neurodevelopmental effects. These findings highlight the need for improved adherence to evidence-based guidelines and further research on the long-term impacts of commonly used neonatal medications as midazolam and dexmedetomidine.

## Supplementary Information

Below is the link to the electronic supplementary material. 

ESM1 (DOCX 30.6 KB)

## Data Availability

The datasets generated during and/or analyzed during the current study are available from the corresponding author upon reasonable request.
