# Correction to: A global cross-sectional survey on neonatal analgosedation: unveiling global trends and challenges through latent class analysis

**DOI:** 10.1007/s00431-025-06100-0

**Published:** 2025-03-26

**Authors:** Cristina Arribas, Giacomo Cavallaro, Nunzia Decembrino, Juan Luis González, Carolina Lagares, Genny Raffaeli, Anne Smits, Sinno P. H. Simons, Eduardo Villamor, Karel Allegaert, Felipe Garrido, on behalf of the ESPR Special Interest Group for Neonatal Pain

**Affiliations:** 1 Neonatal Intensive Care Unit, Clínica Universidad de Navarra, Madrid, Spain; 2 Neonatal Intensive Care Unit, Fondazione IRCCS Ca’ Granda Ospedale Maggiore Policlinico, Milan, Italy; 3 Neonatal Intensive Care Unit, Azienda Ospedaliera Universitaria Policlinico G. Rodolico San Marco, Catania, Italy; 4 Department of Statistics and Operations Research, Faculty of Medicine, University of Cadiz, Cádiz, Spain; 5 Department of Clinical Sciences and Community Health, Università Degli Studi Di Milano, Milan, Italy; 6 Department of Development and Regeneration, KU Leuven, Leuven, Belgium; 7 Neonatal Intensive Care Unit, University Hospitals Leuven, Leuven, Belgium; 8 Department of Pediatric and Neonatal Intensive Care, Division of Neonatology, Erasmus University Medical Center, Sophia Children’s Hospital, Rotterdam, The Netherlands; 9 Division of Neonatology, Mosakids Children’S Hospital, Maastricht University Medical Center (MUMC+), Research Institute for Oncology and Reproduction (GROW), Maastricht University, Maastricht, The Netherlands; 10 Department of Hospital Pharmacy, Erasmus MC, Rotterdam, The Netherlands; 11 Department of Pharmaceutical and Pharmacological Sciences, KU Leuven, Leuven, Belgium

**Correction to: European Journal of Pediatrics (2025) 184:241**

10.1007/s00431-025-06074-z

In the originally published article entitled “A global cross sectional survey on neonatal analgosedation: unveiling global trends and challenges through latent class analysis”, the study group name “ESPR Special Interest Group for Neonatal Pain” has been omitted from the author group.

The original article has been corrected.

