# Supplementary material for: A global cross-sectional survey on neonatal analgosedation: unveiling global trends and challenges through latent class analysis
Source: Eur J Pediatr. 2025 Mar 12;184(4):241. doi: 10.1007/s00431-025-06074-z (PMC11903631; doi:10.1007/s00431-025-06074-z)
Supplement: Supplementary file 1 — (DOCX 30.6 KB) [file 431_2025_6074_MOESM1_ESM.docx]

| **Supplemental Table 1. Members of ESPR Neonatal Pain Special Interest Group** | | |
| --- | --- | --- |
| **Members** | **E-mail (will not be published)** | **Affiliation, City, Country** |
| Abigail Kusi Amponsah | akamponsah.fahs@knust.edu.gh | Department of Nursing, Faculty of Allied Health Sciences, College of Health Sciences, Kwame Nkrumah University of Science and Technology, Kumasi, Ghana.Department of Nursing Science, Faculty of Medicine, University of Turku, Turku, Finland. |
| Agnes van den Hoogen | ahoogen@umcutrecht.nl | Department of Neonatology, Wilhelmina Children’s Hospital, University Medical Centre Utrecht, Utrecht University, Utrecht, The Netherlands. |
| Alexandra Ullsten | alexandra.ullsten@oru.se | Center for Clinical Research, Region Värmland, Karlstad, Sweden.Faculty of Medicine and Health, School of Health Sciences, Örebro University, Örebro, Sweden. |
| Angela Amigoni | angela.amigoni@aopd.veneto.it | Pediatric Intensive Care Unit, Department of Woman’s and Child’s Health, University Hospital of Padova, Padova, Italy |
| Anna Axelin | anmaax@utu.fi | Department of Nursing Science, University of Turku, Turku, Finland. |
| Annakaija Palomaa | annakaija.palomaa@gmail.com | Department of Pediatrics and Adolescence, Oulu University Hospital, Oulu, Finland. |
| Anne Smits | anne.smits@uzleuven.be | 1. Department of Development and Regeneration, KU Leuven, Leuven, Belgium 2. Neonatal Intensive Care Unit, University Hospitals Leuven, Leuven, Belgium |
| Aomesh Bhatt | aomesh.bhatt@paediatrics.ox.ac.uk | Department of Paediatrics, University of Oxford, Oxford, United Kingdom. |
| Arild Rønnestad | aronnest@ous-hf.no | Department of Neonatal Intensive Care, Oslo University Hospital, Oslo, Norway.Institute of Clinical Medicine, University of Oslo, Oslo, Norway. |
| Beatrice Olsson Duse | beatrice.duse@gmail.com | Children and the Youth Clinic Eskilstuna Sweden. |
| Bente Johanne Vederhus | bente.johanne.vederhus@helse-bergen.no | Department of Paediatric and Adolescent Medicine, Haukeland University Hospital, Bergen, Norway. |
| Bente Silnes Tandberg | btandb@vestreviken.no | Department of Paediatric and Adolescent Medicine, Drammen Hospital, Vestre Viken Hospital Trust, Drammen, Norway.Lovisenberg Diaconal University College, Oslo, Norway. |
| Bert Joosten | bert.joosten@mumc.nl | Department of Anesthesiology and Pain Management, Maastricht University Medical Center + , Maastricht, The NetherlandsDepartment of Anesthesiology and Pain Management, Division of Translational Neuroscience, School of Mental Health and Neuroscience (MHeNs), University Maastricht, Maastricht, The Netherlands |
| Caroline Hartley | caroline.hartley@paediatrics.ox.ac.uk | Department of Paediatrics, University of Oxford, Oxford, United Kingdom. |
| Charalampos Kotidis | c.kotidis@liverpool.ac.uk | Department of Women’s and Children’s Health, University of Liverpool, Liverpool Health Partners, Liverpool, United Kingdom.University of Liverpool, Liverpool Womens Hospital, Liverpool, United Kingdom. |
| Charles Christoph Roehr | ccroehr@icloud.com | National Perinatal Epidemiology Unit, Clinical Trials Unit, Oxford Population Health, Medical Sciences Division, University of Oxford, Oxford, United Kingdom.Women and Children’s, Neonatal Intensive Care Unit, Southmead Hospital, North Bristol NHS Trust, Westbury on Trym, Bristol, United Kingdom. |
| Christ-Jan van Ganzewinkel | c.vanganzewinkel@mmc.nl | Neonatal Intensive Care Unit, Maxima Medical Centre, Veldhoven, The Netherlands. |
| Cristina Arribas | carribass@unav.es | Neonatal Intensive Care Unit. Clínica Universidad de Navarra. Madrid. Spain. |
| Daniela Sofia Horta Machado | daniela_sofia_machado@hotmail.com | Neonatal Intensive Care Unit, Imperial College NHS TRUST, London, United Kingdom. |
| Daniëlla Roofthooft | d.roofthooft@erasmusmc.nl | Department of Pediatrics, Division of Neonatology, Erasmus MC Sophia Children’s Hospital, Rotterdam, The Netherlands. |
| Eduardo Villamor | e.villamor@mumc.nl | Division of Neonatology, MosaKids Children’s Hospital, Maastricht University Medical Center (MUMC+), SchoolResearch Institute for Oncology and Reproduction (GROW), Maastricht University, 6202AZ Maastricht, The Netherlands. |
| Elisabeth Norman | elisabeth.norman@med.lu.se | Department of Neonatology, Skåne University Hospital, Lund, SwedenDepartment of Paediatrics, Clinical Sciences, Lund University, Lund, Sweden. |
| Emma Olsson | Emma.Olsson@oru.se | Department of Paediatrics, Faculty of Medicine and Health, Örebro University, Örebro, SwedenFaculty of Medicine and Health, School of Health Sciences, Örebro University, Örebro, Sweden |
| Eugene Dempsey | g.dempsey@ucc.ie | Department of Paediatrics and Child Health, Infant Centre, University College Cork, Cork, Ireland. |
| Evalotte Mörelius | e.morelius@ecu.edu.au | Department of Health, Medicine and Caring Sciences, Linköping University, Linköping, Sweden.School of Nursing and Midwifery, Edith Cowan University, Perth, Australia. |
| Felipe Garrido | fgarridom@unav.es | Neonatal Intensive Care Unit. Clínica Universidad de Navarra. Madrid. Spain |
| Flore Le Marechal | flore.le-marechal@hotmail.fr | Department of Paediatric and Adolescent Medicine, Drammen Hospital, Vestre Viken Hospital Trust, Drammen, Norway. |
| Francesca Sperotto | francesca.sperotto@cardio.chboston.org | Department of Cardiology, Boston Children’s Hospital, Boston, MA, USA.Department of Pediatrics, Harvard Medical School, Boston, MA, USA. |
| Genny Raffaeli | genny.raffaeli@policlinico.mi.it | Neonatal Intensive Care Unit. Fondazione IRCCS Ca’ Granda Ospedale Maggiore Policlinico. Milan. Italy |
| Gerbrich van den Bosch | g.vandenbosch@erasmusmc.nl | Department of Neonatal and Pediatric Intensive Care, Division of Neonatology, Erasmus University Medical Center - Sophia Children’s Hospital, Rotterdam, The Netherlands |
| Giacomo Cavallaro | giacomo.cavallaro@policlinico.mi.it | Neonatal Intensive Care Unit. Fondazione IRCCS Ca’ Granda Ospedale Maggiore Policlinico. Milan. Italy |
| Guðrún Kristjánsdóttir | gkrist@hi.is | Faculty of Nursing and Midwifery, University of Iceland, Reykjavik, Iceland.Children´s Hospital, Landspitali University Hospital, Reykjavik, Iceland |
| Hanna Ahl | hanna_ahl@hotmail.com | Department of Neonatology, Skåne University Hospital, Lund, Sweden. |
| Hans Jørgen Stensvold | hstensvo@ous-hf.no | Department of Neonatal Intensive Care, Clinic of Paediatric and Adolescent Medicine, Oslo University Hospital, Oslo, Norway. |
| Helle Haslund-Thomsen | hht@rn.dk | Clinical Nursing Research Unit, Aalborg University Hospital, Aalborg, Denmark.Department of Paediatrics, Aalborg University Hospital, Department of Clinical Medicine, Aalborg University, Aalborg, Denmark |
| Janne Weiss | janne.weis@regionh.dk | Department of Neonatology, The Juliane Marie Centre, Copenhagen University Hospital, Copenhagen, Denmark. |
| Jannicke Hanne Andresen | jandrese@ous-hf.no | Department of Neonatology, Oslo University Hospital, Oslo, Norway. |
| Jean-Michel Roue | jean-michel.roue@chu-brest.fr | Department of Neonatal Medicine, University Hospital of Brest, Brest, France. |
| Joke Wielenga | j.wielenga@amsterdamumc.nl | IC Neonatology Emma Children’s Hospital Amsterdam University Medical Center, Amsterdam, The Netherlands |
| Judith ten Barge | j.tenbarge@erasmusmc.nl | Department of Pediatrics, Division of Neonatology, Sophia Children’s Hospital, Rotterdam, The Netherlands. |
| Karel Allegaert | karel.allegaert@uzleuven.be | 1. Department of Development and Regeneration, KU Leuven, Leuven, Belgium. 2. Department of Hospital Pharmacy, Erasmus MC, Rotterdam, The Netherlands 3. Department of Pharmaceutical and Pharmacological Sciences, KU Leuven, Leuven, Belgium |
| Katrin Klebermass-Schrehof | katrin.klebermass-schrehof@meduniwien.ac.at | Division of Neonatology, Pediatric Intensive Care and Neuropediatrics, Department of Pediatrics and Adolescent Medicine, Comprehensive Center for Pediatrics, Medical University Vienna, Austria |
| Laila Kristoffersen | laila.kristoffersen@ntnu.no | Department of Neonatology, St Olavs Hospital Trondheim University Hospital, Trondheim, Norway.Department of Public Health and Nursing, Norwegian University of Science and Technology, Trondheim, Norway. |
| Laura Moschino | lauramoschino13@gmail.com | Neonatal Intensive Care Unit, Padova University Hospital, Padova, Italy |
| Lene Lyngstad | lene.tandle@vestreviken.no | Department of Paediatric and Adolescent Medicine, Drammen Hospital, Vestre Viken Hospital Trust, Drammen, Norway. |
| Liam Mahoney | l.mahoney@doctors.org.uk | University Hospitals Bristol and Weston NHS Foundation Trust, Bristol, United Kingdom.Regional Neonatal Intensive Care Unit St. Michael’s Hospital, Bristol, United Kingdom. |
| Luke Baxter | luke.baxter@paediatrics.ox.ac.uk | Department of Paediatrics, University of Oxford, Oxford, United Kingdom. |
| Magdalena Panek | panek.magdalena.mp@gmail.com | Department of Maternal and Child Health, Faculty of Health Sciences, Jagiellonian University Medical College, Krakow, Poland. |
| Manon Tauzin | manon.tauzin@chicreteil.fr | Neonatal Intensive Care Unit, Centre Hospitalier Intercommunal de Créteil, Créteil, France. |
| Maria Gradin | maria.gradin@regionorebrolan.se | Department of Pediatrics, Faculty of Medicine and Health, Örebro University, Örebro, Sweden. |
| Mariaana Mäki-Asiala | mariaana.maki-asiala@student.oulu.fi | Research Unit of Health Sciences and Technology, Faculty of Medicine, University of Oulu, Oulu, Finland.Medical Research Center Oulu, Oulu University Hospital, University of Oulu, Oulu, Finland. |
| Marsha Campbell-Yeo | marsha.campbell-yeo@dal.ca | School of Nursing, Faculty of Health, Departments of Pediatrics, Psychology and Neuroscience, Dalhousie University and IWK Health, Halifax, Nova Scotia, Canada |
| Martina Carlsen Misic | martina.carlsen.misic@oru.se | Department of Pediatrics, Faculty of Medicine and Health, Örebro University, Örebro, Sweden.Faculty of Medicine and Health, School of Health Sciences, Örebro University, Örebro, Sweden. |
| Mathilde Baudat | m.baudat@maastrichtuniversity.nl | Department of Anesthesiology and Pain Management, Maastricht University Medical Centre+, Maastricht, The NetherlandsDepartment of Translational Neuroscience, School of Mental Health and Neuroscience, Maastricht University, Maastricht, The Netherlands. |
| Mats Eriksson | mats.h.eriksson@oru.se | School of Health Sciences, Faculty of Medicine and Health, Örebro University, Örebro, Sweden |
| Máximo Vento | maximo.vento@uv.es | Neonatal Research Group at the Health Research Institute La Fe (IISLAFE), Valencia, Spain. |
| Monique van Dijk | m.vandijk.3@erasmusmc.nl | Department of Neonatal and Pediatric Intensive Care, Division of Pediatric Intensive Care, Erasmus MC-Sophia Children’s Hospital, Erasmus University Medical Center Rotterdam, Rotterdam, The Netherlands.Department of Internal Medicine, Division of Nursing Science, Erasmus MC, Erasmus University Medical Center Rotterdam, Rotterdam, The Netherlands. |
| Naomi Meesters | n.meesters@erasmusmc.nl | Department of Neonatal and Pediatric Intensive Care, Division of Neonatology, Erasmus MC –Sophia Children’s Hospital, Rotterdam, the Netherlands |
| Natalia Toumbourou | natalia.toumbourou@wh.org.au | Neonatal Intensive Care Unit, Western Health Hospital, Melbourne, Australia |
| Nunzia Decembrino | n.decembrino@policlinico.unict.it | Neonatal Intensive Care Unit, AOU Policlinico G. Rodolico San Marco, Catania, Italy |
| Paola Lago | paola.lago@sanita.padova.it | Neonatal Intensive Care Unit, Department of Critical Care, Cà Foncello Regional Hospital, Treviso, Italy. |
| Pia Lundqvist | pia.lundqvist@med.lu.se | Department of Health Sciences, Lund University, Lund, Sweden. |
| Randi Dovland Andersen | anrd@sthf.no | Department of Research, Telemark Hospital Trust, Skien, NorwayResearch Centre for Habilitation and Rehabilitation Models & Services (CHARM), The Faculty of Medicine, University of Oslo, Oslo, Norway |
| Rebeccah Slater | rebeccah.slater@paediatrics.ox.ac.uk | Department of Paediatrics, University of Oxford, Oxford, United Kingdom. |
| Ricardo Carbajal | ricardo.carbajal@aphp.fr | Pediatric Emergency Department, Assistance Publique-Hôpitaux de Paris, Hôpital Armand Trousseau-Sorbonne Université, Paris, France.Institut National de La Santé et de La Recherche Médicale, Paris, France. |
| Rikke Louise Stenkjær | rikke.louise.stenkjaer@regionh.dk | Department of Intensive Care for Infants and Toddlers, Copenhagen University Hospital Rigshospitalet, Copenhagen, Denmark. |
| Robert B. Flint | r.flint@erasmusmc.nl | Department of Hospital Pharmacy, Erasmus University Medical Center, Rotterdam, The NetherlandsDepartment of Pediatrics, Division of Neonatology, Erasmus University Medical Center - Sophia Children’s Hospital, Rotterdam, The Netherlands |
| Samir El Abdouni | s.elabdouni@erasmusmc.nl | Department of Hospital Pharmacy, Erasmus University Medical Center, Rotterdam, The Netherlands |
| Scott Montgomery | scott.montgomery@oru.se | Clinical Epidemiology and Biostatistics, Department of Medical Sciences, Faculty of Medicine and Health, Örebro University, Sweden.Clinical Epidemiology Division, Department of Medicine, Solna, Karolinska Institute, Stockholm, Sweden.Department of Epidemiology and Public Health, University College London, London, United Kingdom. |
| Serdar Beken | serbeken@gmail.com | Department of Pediatrics, Section of Neonatology, Acibadem Mehmet Ali Aydinlar University, School of Medicine, İstanbul, Turkey. |
| Sezin Ünal | sezinunal@gmail.com | Division of Neonatology, Baskent University Faculty ofMedicine, Ankara, Turkey. |
| Shalini Ojha | Shalini.Ojha@nottingham.ac.uk | Centre for Perinatal Research, School of Medicine, University of Nottingham, Nottingham, United Kingdom.Neonatal Unit, University Hospitals of Derby and Burton NHS Trust, Derby, United Kingdom. |
| Shellie Robinson | shellie.robinson@ouh.nhs.uk | Department of Paediatrics, University of Oxford, Oxford, United Kingdom. |
| Sigríður María Atladóttir | sigmaa@landspitali.is | Faculty of Nursing, University of Iceland, Reykjavik, Iceland.Neonatal Intensive Care Unit, Lanspitali University Children’s Hospital, Reykjavik, Iceland. |
| Sinno HP Simons | s.simons@erasmusmc.nl | Department of Pediatrics, Division of Neonatology, Erasmus University Medical Center - Sophia Children’s Hospital, Rotterdam, The Netherlands |
| Sofie Pirlotte | Sofie.Pirlotte@uzbrussel.be | Neonatal Intensive Care Unit, University UZ Brussel, Brussels, Belgium |
| Solfrid Steinnes | solfrid.steinnes@ldh.no | Department of Paediatric and Adolescent Medicine Neonatal Intensive Care Unit Drammen Hospital Vestre Viken Hospital Trust Drammen Norway. |
| Swantje Völler | s.voller@lacdr.leidenuniv.nl | Division of Systems Pharmacology and Pharmacy, Leiden Academic Centre for Drug Research, Leiden University, Leiden, The Netherlands. |
| Tarja Pölkki | tarja.polkki@oulu.fi | Research Unit of Health Sciences and Technology, University of Oulu, Oulu, FinlandMedical Research Center Oulu, Oulu University Hospital and University of Oulu, Oulu, Finland |
| Tiina Ukkonen | ukkonen.tiina@gmail.com | 1. Department of Pediatrics and Adolescent Medicine, Oulu University Hospital, Oulu, Finland. 2. Medical Research Center Oulu, Oulu University Hospital and University of Oulu, Oulu, Finland. |
| Tom Stiris | tom.stiris@medisin.uio.no | Department of Neonatal Intensive Care, Oslo University Hospital, Oslo, Norway.Institute of Clinical Medicine, University of Oslo, Oslo, Norway. |
| Xavier Durrmeyer | xavier.durrmeyer@chicreteil.fr | Neonatal Intensive Care Unit, CHI Créteil, Créteil, France. |
